# Drought Severity and Nitrogen Addition Interactively Modulate Seedling Growth and Resource-Use Strategies of *Quercus wutaishanica*

**DOI:** 10.3390/biology15130991

**Published:** 2026-06-24

**Authors:** Qinghua Yang, Huling Zhang, Jiazhi Wang, Jiming Cheng, Hong Ma, Haili Wang, Yonghong Luo

**Affiliations:** 1 School of Biological Science and Engineering, North Minzu University, Yinchuan 750021, China; yqh9962@163.com (Q.Y.); zhanghuling2026@163.com (H.Z.); 13037955817@163.com (H.M.); 13299572026@163.com (H.W.); 2 Chengde Meteorological Disaster Prevention Center of Hebei Province, Chengde 067000, China; george0602@126.com

**Keywords:** drought stress, functional traits, nitrogen, photosynthetic indices, physiological indices

## Abstract

Global climate change has intensified drought and increased nitrogen deposition, threatening forest tree seedling regeneration. To clarify how drought severity and nitrogen enrichment jointly affect seedling performance in *Quercus wutaishanica*, a dominant montane tree in northern China, we conducted a full two-factor pot experiment. We established three drought treatments (ambient precipitation [CK], chronic drought [CD], and intense drought [ID]), fully crossed with two nitrogen addition levels (0 and 10 g N m^−2^ yr^−1^), and measured functional traits related to growth, photosynthesis, and stress resistance. Our main results were threefold: (1) Both drought treatments significantly inhibited growth and biomass accumulation: total biomass decreased by 28% under CD and 38% under ID relative to CK, with suppression intensifying as drought severity increased. (2) Nitrogen addition increased total biomass by 12% under chronic drought, but this ameliorative effect fell to just 2% under intense drought. (3) As drought stress increased, the seedlings underwent a shift from stomatal to non-stomatal photosynthetic limitation, and from active physiological acclimation to irreversible metabolic damage. (4) Random forest modeling confirmed that biomass variation was primarily driven by traits related to water-use efficiency and resource acquisition. Overall, intensifying drought consistently constrains *Q. wutaishanica* seedling growth, and the beneficial effect of nitrogen addition declines sharply with increasing drought severity. These findings provide new empirical insights for predicting seedling regeneration and guiding sustainable forest nutrient management under ongoing climate change.

## Simple Summary

Global climate change has led to more frequent droughts and elevated atmospheric nitrogen deposition in northern China, threatening local *Quercus wutaishanica* seedlings and their reproductive capacity. We investigated how different drought regimes and supplemental nitrogen influenced the growth of these seedlings. Intense drought markedly slowed seedling growth. Nitrogen addition promoted seedling growth under chronic drought, yet this positive effect vanished under intense drought. This study identified the threshold for the interactive effects of drought and nitrogen. The findings provide references for protecting *Q. wutaishanica* forests, restoring local vegetation, optimizing forest nutrient management, and predicting how these seedlings will adapt to future climate change.

## 1. Introduction

Global warming has increased the frequency, duration, and severity of extreme droughts [1,2]. Meanwhile, accelerated atmospheric nitrogen (N) deposition, driven by fossil fuel combustion and excessive fertilizer application, has made China a global hotspot for N deposition [3]. Chronic exogenous N inputs substantially reshape core plant metabolic pathways [4,5]. The interactive effects of drought and N deposition constitute critical constraints on seedling survival and natural regeneration in temperate forests. Elucidating the underlying mechanisms is critical for developing climate change adaptation strategies.

Drought stress impairs seedling survival by inducing water deficit, photosynthetic suppression, membrane lipid peroxidation, and disordered osmotic regulation [6]. Existing studies have confirmed that plants can acclimate to stress via physiological and biochemical pathways, yet excessive stress intensity disrupts protective systems and induces irreversible damage or mortality [7,8]. Most studies have focused on a single drought level, whereas few have compared chronic versus intense drought, limiting our understanding of plant response patterns from acclimation to injury.

Nitrogen is a critical nutrient regulating drought tolerance and plant adaptation to dry environments [9]. Insufficient N availability suppresses plant growth, reduces photosynthetic efficiency, and accelerates leaf senescence [10]. Accordingly, N fertilizer is widely applied to enhance plant stress tolerance [11]. Moderate N inputs can alleviate drought-induced growth constraints [12] and promote biomass accumulation in seedlings under moderate drought stress [13]. Nevertheless, under severe or extreme drought stress, exogenous N is poorly taken up and utilized by plants [14,15]. Most existing studies on drought and nitrogen have focused on crops and herbaceous species, with limited systematic evidence available for tree seedlings in warm-temperate forests exposed to combined drought and nitrogen addition treatments.

*Quercus wutaishanica* is a dominant constructive species in warm-temperate deciduous broad-leaved forests in China, with key functions in soil and water conservation and biodiversity maintenance [16]. Most studies have focused on single-factor responses, and few systematic two-factor experiments have investigated the coupling of drought intensity and N addition. In particular, the threshold responses, physiological mechanisms, and adaptive strategies underlying N-mediated drought stress mitigation under intense drought remain unclear. This knowledge gap hinders accurate evaluation of seedling regeneration limitations under climate change and restricts the development of science-based precision tending and nitrogen management strategies. Addressing these research gaps and practical demands for regional forest natural regeneration, this study aimed to answer two core research questions: (1) Does intense drought impose a significantly stronger inhibitory effect on *Q. wutaishanica* seedlings than chronic drought? (2) Does the mitigating effect of nitrogen addition on drought stress vary with drought intensity?

## 2. Materials and Methods

### 2.1. Seed Collection and Processing

**Figure 1:**
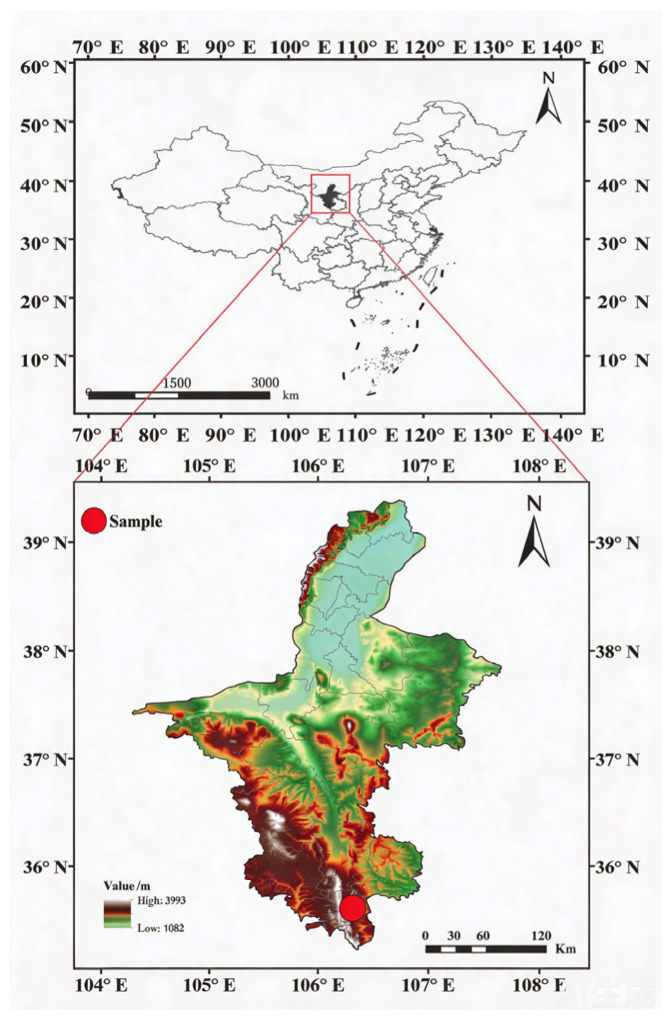
Location of the study area.

Seeds were collected in September 2024 from mature *Q. wutaishanica* growing in Longtan Forest Farm of Liupanshan National Nature Reserve, Ningxia Hui Autonomous Region, China (35°15′–35°41′ N, 106°09′–106°30′ E). The specific sampling location is clearly marked with a red dot in Figure 1. The study area has an annual precipitation of 600 mm and a mean annual temperature of 5.8 °C, with rainfall mainly concentrated in June to September. The soil is mainly cinnamon soil with small patches of red soil [16]. The collected seeds were washed with distilled water, and then we selected plump, insect-free, and intact healthy seeds, which were then stored in breathable self-sealing bags at −4 °C for pretreatment.

### 2.2. Sowing, Seedling Rearing and Transplanting

The pretreated seeds were sown in uniform plastic pots (12 cm in diameter and 13 cm in height) in early October 2024. The main physicochemical properties of the substrate were as follows: soil bulk density, 0.88 g cm^−3^; pH, 6.35; cation exchange capacity (CEC), 32.9 cmol kg^−1^; and electrical conductivity (EC), 0.09 mS cm^−1^ [17]. After being fully homogenized, the soil was evenly apportioned into each pot. Pots were moved into an artificial greenhouse for overwintering at 5–15 °C under natural light, with regular watering to maintain substrate moisture. In mid-April 2025, seedlings were transferred to a rain-out shelter and acclimated for 2 weeks to ensure uniformity before the formal experiment. The formal experiment ran from May to October 2025. Samples for photosynthetic parameters and enzyme activities were collected and fixed in mid-August (10 months after sowing). Seedlings with intact roots were harvested and fixed in late October for growth index determination (12 months after sowing).

### 2.3. Experimental Design

A two-factor randomized block design was employed, with two treatment factors: drought treatment and nitrogen addition.

All treatments were fully combined, forming a total of six treatment groups with seven replicates per group. Fifteen seeds were sown per pot. The total seed consumption was 15 seeds/pot × 7 replicates × 6 treatments = 630 seeds. During the experimental period, natural rainfall was excluded by a rain-out shelter.

Nitrogen addition treatment: Two levels, N0 (0 g N m^−2^ yr^−1^) and N10 (10 g N m^−2^ yr^−1^), applied in early May. The N application was set based on the estimated atmospheric N deposition level in northern China, which can reach 8.5 g N m^−2^ yr^−1^, and model projections suggest that this value will continue to increase in the future [18]. An amount of 10 g N m^−2^ yr^−1^ was adopted as the nitrogen addition rate in several previous studies [12]. Powdered ammonium nitrate was used for nitrogen addition. The application rate per pot was calculated as 0.241 g according to the surface area of pots with a diameter of 12 cm. The fertilizer was fully dissolved in 500 mL of water and sprayed uniformly. An equal volume of water was applied to the control group to ensure consistent moisture levels.

**Figure 2:**
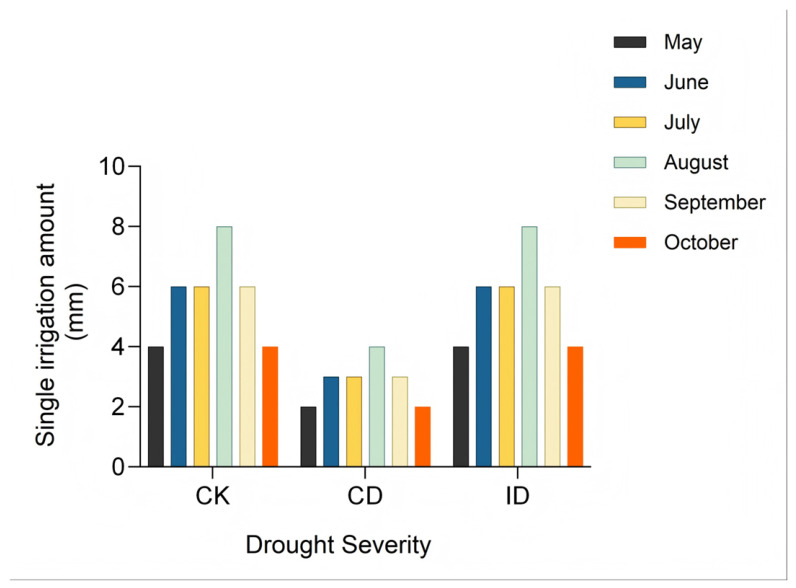
Individual irrigation volume (mm) for *Q. wutaishanica* seedlings across May–October under contrasting drought treatments. CK, CD, and ID indicate natural precipitation, chronic drought, and intense drought.

Drought treatment: Three levels, natural precipitation (CK), 50% reduction in each precipitation event (chronic drought, CD), and complete rain exclusion for 7 days per month with a normal rainfall amount and interval for the rest of the month (intense drought, ID). The rainfall interval was fixed at two days. Drought treatments were conducted from May to October. Monthly irrigation amounts and intervals were calculated based on 40-year local precipitation records (Table S1, Figure 2).

### 2.4. Measurement of Experimental Variables

#### 2.4.1. Measurement of Morphological Traits

At the end of October 2025, surviving seedlings were collected from each treatment. Seedlings were sealed in bags and transported to the laboratory. Roots and leaves were rinsed to remove surface soil, blotted dry, and weighed for fresh biomass of leaves, stems, and roots. Leaf area (LA, cm^2^) was measured using the grid-paper method. Tissues were then oven-dried at 85 °C for 48 h, and the leaf dry weight (LDW), root dry weight (RDW), and stem dry weight (SDW) were determined. Three pots were randomly selected for each treatment. For each pot containing multiple seedlings, the average value of all measurements was calculated. Specific leaf area (SLA, cm^2^/g), root–shoot ratio (R/S), and total biomass (TB, g) were calculated using standard formulas described by [19].

SLA (cm^2^/g) = LA (cm^2^)/LDW (g) (1)

R/S = RDW (g)/(LDW (g) + SDW (g))(2)

TB(g) = LDW(g) + SDW (g) + RDW(g)(3)

#### 2.4.2. Measurement of Photosynthetic Parameters

In mid-August 2025, on sunny mornings (9:00–11:00), a portable photosynthesis system (LI-6400XT, LI-COR Biosciences, Lincoln, NE, USA) was used to measure net photosynthetic rate (Pn), stomatal conductance (Gs), intercellular CO_2_ concentration (Ci), transpiration rate (Tr), and instantaneous water-use efficiency (iWUE). To quantify chlorophyll (Chl) content, three pots were randomly chosen per treatment, with five leaves collected from each pot for analysis, and mean values were computed for each pot. All leaf samples were sealed and refrigerated under dark conditions at 4 °C. Chl was extracted from 0.1 g of fresh leaves using 80% acetone. The absorbance at 645 nm and 663 nm was detected, and relevant indices were calculated following Li H. [20].

iWUE = Pn/Tr(4)

Chl a = 12.72 × A_663_ − 2.59 × A_645_ (5)

Chl b = 22.88 × A_645_ − 4.67 × A_663_ (6)

Chl = 20.29 × A_645_ + 8.05 × A_663_ (7)

#### 2.4.3. Determination of Enzymatic Parameters and Osmotic Adjustment Substances

In mid-August 2025, three pots were randomly selected from each treatment. Five leaves were sampled from each pot, and the mean value of the measurements was calculated. After collection, all leaf samples were immediately frozen in liquid nitrogen, then sealed and stored in the dark at −20 °C. The leaf samples were cut (0.1 g) and homogenized for physiological assays. Catalase (CAT) activity was tested by ultraviolet absorption: after mixing the homogenate with hydrogen peroxide, the absorbance at 240 nm was monitored for 3 min. For malondialdehyde (MDA) analysis, the extract was incubated with thiobarbituric acid (TBA) reagent in a boiling water bath, and the absorbance at 450 nm, 532 nm and 600 nm was read after cooling. Soluble protein (SP) content was assessed using the Coomassie Brilliant Blue G-250 method, and the absorbance at 595 nm was recorded. Proline (Pro) reacted with ninhydrin under acidic heating conditions, followed by toluene extraction and absorbance measurement at 520 nm [20].

### 2.5. Statistical Analyses

First, two-way analysis of variance (ANOVA) was used to examine the effects of drought regimes (CK vs. CD vs. ID) and N addition levels (N0 vs. N10) on plant growth traits (LDW, RDW, SDW, SLA, R/S, TB), leaf gas exchange parameters (Pn, Gs, Ci, Tr, iWUE), Chl content, and physiological and biochemical indices (CAT, MDA, SP, Pro). All data are presented as the mean ± standard deviation (Mean ± SD). Drought treatment and nitrogen addition were set as fixed effects in these models to verify their independent effects.

Subsequently, after model validation to satisfy the assumptions of normality, homogeneity of variance, and residual independence, post hoc analyses were performed to identify specific intergroup differences. Tukey’s honestly significant difference (HSD) test via the emmeans package (v1.8.9) was adopted for pairwise comparisons among drought treatments, and significant differences (*p* < 0.05) were marked with different uppercase letters in corresponding figures and tables. The paired *t*-test was used to compare different nitrogen levels (N0 vs. N10) under the same drought condition, with significant differences (*p* < 0.05) denoted by lowercase letters.

Finally, to further clarify the relative explanatory power of seedling growth-related parameters and treatments on the total biomass of *Q. wutaishanica* seedlings, a random forest model was established using the randomForest package (v4.7-1.1). The model parameters were set as follows: the number of decision trees (ntree) = 500, and the number of variables randomly sampled at each split (mtry) was set as the default value. Tenfold cross-validation was conducted to ensure model reliability and stability. Variable importance was ranked according to the percentage increase in mean squared error (%IncMSE), and permutation tests were applied to verify the significance of variable contributions.

## 3. Results

### 3.1. Effects of Drought and Nitrogen Addition on Seedling Growth

**Figure 3:**
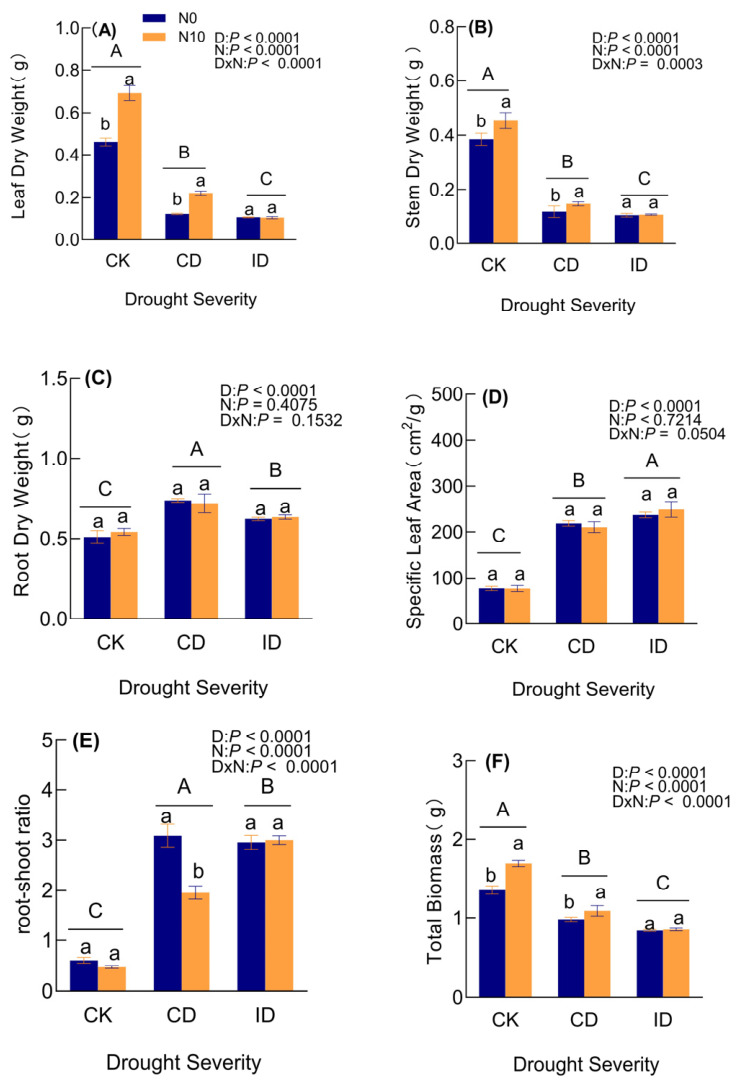
Effects of nitrogen addition and drought on growth traits of *Q. wutaishanica* seedlings. (**A**) Leaf dry weight (g); (**B**) Stem dry weight (g); (**C**) Root dry weight (g); (**D**) Specific leaf area (cm^2^ g^−1^); (**E**) Root-shoot ratio; (**F**) Total biomass (g). Values are shown as mean ± standard deviation. CK, CD, and ID indicate natural precipitation, chronic drought, and intense drought. D, N and D×N denote *p*-values for drought, nitrogen and drought–nitrogen interaction, respectively. Significant differences among drought treatments are marked by different uppercase letters, while different lowercase letters indicate significant differences among nitrogen levels within the same drought treatment (*p* < 0.05). Full raw source data supporting this figure are provided in Table S2.

Compared with CK, drought treatments significantly reduced LDW, RDW, and TB, with stronger reductions under ID than CD. By contrast, RDW, SLA, and R/S increased significantly under drought conditions (Figure 3A,B,F). N addition significantly alleviated drought stress under CD but had little effect under ID. Drought and N addition showed significant interactive effects on LDW, SDW, R/S, and TB (Figure 3A,B,E,F).

### 3.2. Effects on Photosynthetic Physiology

**Figure 4:**
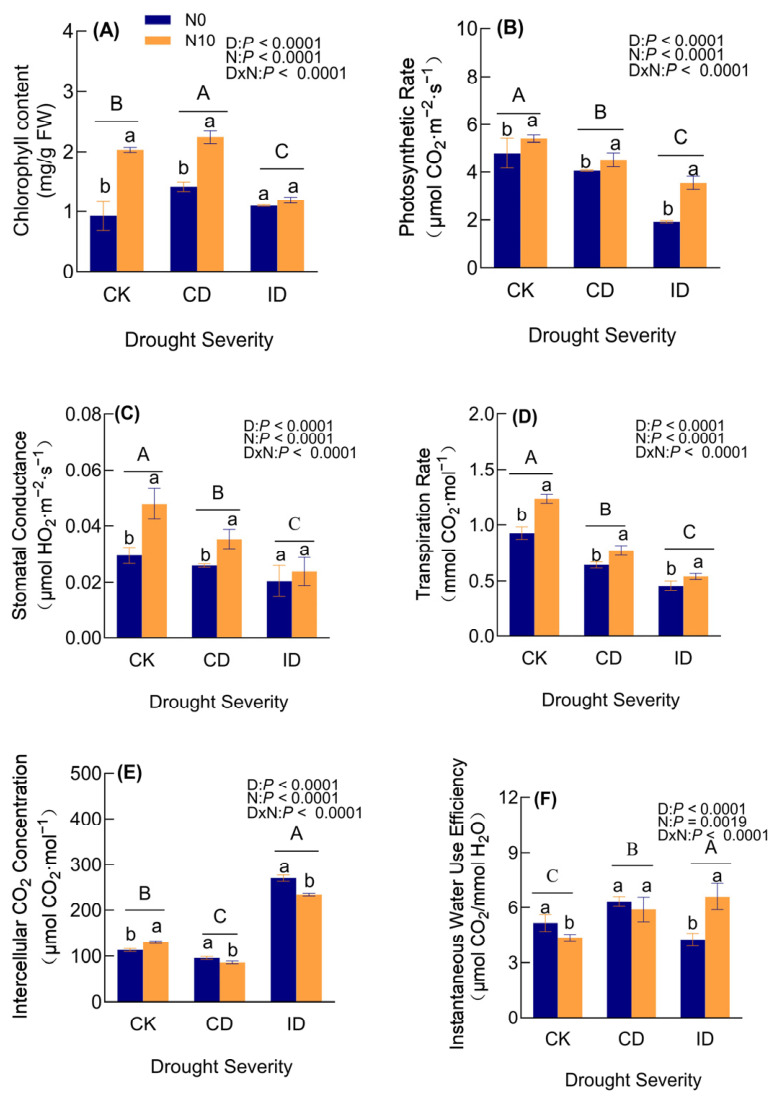
Effects of nitrogen addition and drought on the photosynthetic physiology of *Q. wutaishanica* seedlings. (**A**) Chlorophyll content (mg g^−1^ FW); (**B**) Photosynthetic rate (μmol CO_2_·m^−2^·s^−1^); (**C**) Stomatal conductance (μmol H_2_O·m^−2^·s^−1^); (**D**) Transpiration rate (mmol CO_2_·mol^−1^); (**E**) Intercellular CO_2_ concentration (μmol CO_2_·mol^−1^); (**F**) Instantaneous water use efficiency (μmol CO_2_/mmol H_2_O). Values are shown as mean ± standard deviation. CK, CD, and ID indicate natural precipitation, chronic drought, and intense drought. D, N and D×N denote *p*-values for drought, nitrogen and drought–nitrogen interaction, respectively. Significant differences among drought treatments are marked by different uppercase letters, while different lowercase letters indicate significant differences among nitrogen levels within the same drought treatment (*p* < 0.05). Full raw source data supporting this figure are provided in Table S2.

Drought significantly decreased Pn, Gs, and Tr, with larger reductions under ID than CD, while iWUE increased significantly (Figure 4B–D,F). Under CD, Chl increased and Ci decreased significantly; under ID, Chl decreased sharply and Ci increased markedly (Figure 4A,E). N addition effectively mitigated photosynthetic inhibition under CD but not under ID treatment. Drought and N addition interacted significantly on all photosynthetic indices (Figure 4A–F).

### 3.3. Effects on Physiological and Biochemical Traits

**Figure 5:**
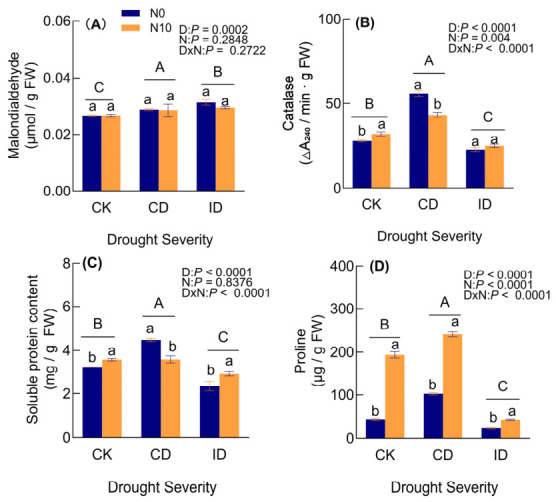
Effects of nitrogen addition and drought on physiological and biochemical characteristics of *Q. wutaishanica* seedlings. (**A**) Malondialdehyde concentration (μmol g^−1^ FW); (**B**) Catalase (ΔA_240_ min^−1^ g^−1^ FW); (**C**) Soluble protein content (mg g^−1^ FW); (**D**) Proline (μg g^−1^ FW). Values are shown as mean ± standard deviation. CK, CD, and ID indicate natural precipitation, chronic drought, and intense drought. D, N and D×N denote *p*-values for drought, nitrogen and drought–nitrogen interaction, respectively. Significant differences among drought treatments are marked by different uppercase letters, while different lowercase letters indicate significant differences among nitrogen levels within the same drought treatment (*p* < 0.05). Full raw source data supporting this figure are provided in Table S2.

Under CD, MDA, SP, Pro, and CAT activity increased significantly; under ID, all these indices decreased markedly (Figure 5A–D). N addition significantly alleviated physiological changes under CD but not under ID treatment. Drought and N addition exhibited significant interactive effects on the CAT, SP, and Pro of seedlings (Figure 5A–D).

### 3.4. Explanatory Power of Seedling-Growth-Related Parameters and Treatments for Total Biomass

**Figure 6:**
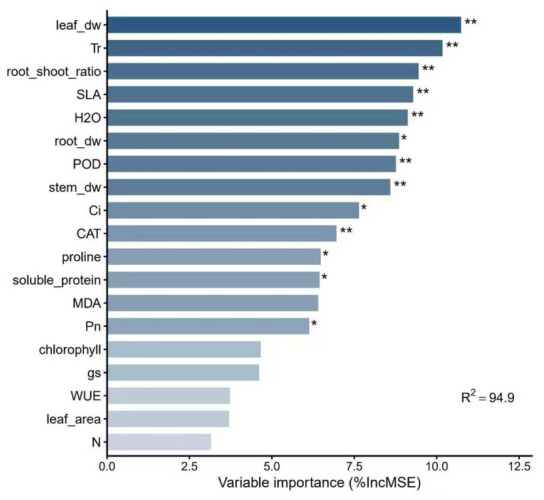
Contributions of seedling growth parameters and experimental treatments to total biomass. Note: leaf_dw = leaf dry weight, Tr = transpiration rate, root_shoot_ratio = root–shoot ratio, SLA = specific leaf area, H_2_O = drought treatment, root_dw = root dry weight, POD = peroxidase, stem_dw = stem dry weight, Ci = intercellular CO_2_ concentration, CAT = catalase, proline = proline content, soluble_protein = soluble protein, MDA = malondialdehyde, Pn = net photosynthetic rate, chlorophyll = chlorophyll content, gs = stomatal conductance, WUE = water-use efficiency, leaf_area = leaf area, N = nitrogen treatment. Mean squared error (MSE) was used to quantify variable importance. Asterisks (*, **) indicate significant explanatory effects of independent variables on the dependent variable (* *p* < 0.05, ** *p* < 0.01). Full raw source data supporting this figure are provided in Table S2.

The model had a high coefficient of determination (R^2^ = 94.9), explaining 94.9% of total biomass variation. The most influential variables in descending order were: LDW, Tr, R/S, SLA, soil water, RDW, POD, SDW, CAT, Pro, and SP. All these variables significantly explained total biomass (* *p* < 0.05, ** *p* < 0.01) (Figure 6).

## 4. Discussion

### 4.1. Drought Intensity Effect: Intense Drought Causes Stronger Inhibition and Physiological Collapse

The results of this study indicated that both drought intensities induced the collapse of overall plant metabolic functions. Compared with chronic drought, intense drought exerted significantly stronger inhibitory effects on *Q. wutaishanica* seedlings, showing a typical intensity-dependent response pattern. This finding addresses our first research question. Drought response patterns of *Q. wutaishanica* seedlings conform to the stress-gradient hypothesis [21,22]. Adaptive regulation dominates under low stress, while physiological damage prevails under high stress. Beyond a critical threshold, systemic functional failure occurs [23,24]. Collectively, this evidence verifies that extreme drought acts as a key disturbance limiting the natural regeneration of forest communities [25].

Drought-driven inhibition of seedling biomass accumulation became more pronounced as drought severity rose. Under chronic drought, the R/S increased, and biomass was preferentially allocated to roots, consistent with global meta-analyses [19] and optimal resource allocation theory: plants prioritize resource acquisition organs to capture limited water [26,27]. Increased SLA reflects a resource-acquisitive strategy to maintain carbon gain at low cost [28,29,30]. Plants have sufficient time for morphological adjustment under chronic drought, exhibiting an obvious growth–survival trade-off strategy [31,32]. In contrast, under intense drought, plants suffer severe physiological damage with suppressed photosynthesis and severely insufficient resource supply. This adaptive strategy collapsed, and seedling biomass growth was drastically restricted.

At the photosynthetic physiological level, the inhibition of photosynthesis by drought gradually shifts from stomatal limitation to non-stomatal limitation [33,34]. Under chronic drought, elevated Chl, stomatal closure, and reduced Ci indicate active stomatal regulation and homeostasis [7]. Under intense drought, chloroplast membrane damage, Chl degradation, and reduced mesophyll capacity reflect non-stomatal limitation and irreversible damage [35]. Increased iWUE reflects a water-saving, conservative strategy under water limitation [33,36].

From the stress physiological perspective, *Q. wutaishanica* seedlings exhibited an obvious shift in drought response: from mild oxidative stress to severe antioxidant dysfunction. Chronic drought increases CAT activity and SP and Pro concentrations, enhancing antioxidant defense, osmotic adjustment, and membrane stability [37]. Under intense drought, excessive reactive oxygen species surpass the antioxidant defense capacity. CAT activity drops sharply, MDA accumulates greatly, and the production of osmoprotectants is inhibited, indicating disrupted homeostasis and metabolic failure [38]. Accordingly, seedlings transition from active physiological acclimation to passive damage, with their regulatory strategies replaced by suppressed physiological function under intense drought stress.

### 4.2. Nitrogen Mitigation Depends on Drought Intensity: Effective Under Chronic Drought, Ineffective Under Intense Drought

Our findings revealed a stress threshold, which answers the second research question: N addition mitigates drought only under chronic drought (moderate drought), not under intense drought. This aligns with the results of many previous studies [12,13]. It can be seen that water and N are two core limiting factors for plant growth, and their coupling effect follows the resource limitation hierarchy theory. When one resource is extremely scarce, the regulatory effect of the other is completely masked [39,40].

Under moderate drought, without absolute water limitation, N improves drought tolerance via coordinated trait regulation [41]: increasing Chl and photosynthetic enzymes to relieve stomatal limitation; strengthening antioxidant systems; reducing lipid peroxidation; enhancing osmotic adjustment; and maintaining cellular homeostasis. Our results indicate that under moderate drought, the coordinated regulation induced by N addition is accompanied by a significant reduction in the root–shoot ratio, representing an adaptive resource allocation strategy [6]. When N is sufficient, seedlings are no longer constrained by nutrient availability and instead allocate resources to aboveground growth and photosynthesis [41]. Elevated proline helps scavenge reactive oxygen species and maintain cell turgor, while stable malondialdehyde levels suggest reduced lipid peroxidation and improved membrane integrity [38]. In this case, nitrogen becomes the main limiting nutrient, and coordinated adjustments in biomass allocation, photosynthesis, and stress resistance effectively improve seedling drought tolerance.

N efficacy is diminished under intense drought, and such a mechanism is consistent with the resource bottleneck theory [42,43]. Severe soil water deficit impairs root water uptake pathways and upward transport [44], preventing N absorption, translocation, and assimilation. Exogenous N cannot enter metabolic pathways for Chl, protein, or enzyme synthesis [4,5]. Meanwhile, water scarcity induces massive reactive oxygen species accumulation, organelle damage, and irreversible membrane impairment, leading to systemic metabolic collapse [6]. Even with added N, water limitation fully blocks N utilization. Thus, our results demonstrate that nitrogen addition provides no significant benefit to seedlings under intense drought conditions.

### 4.3. Key Driving Factors of Total Biomass

Random forest analysis quantified trait contributions to biomass. The results showed that traits related to water-use efficiency and biomass allocation (resource-acquisition capacity) had the highest explanatory power, confirming that seedling growth under drought is governed mainly by biomass partitioning and internal water homeostasis [31,45]. LDW, Tr, R/S, SLA, and soil water content collectively explained over 70% of the variance in total biomass. These traits align with the plant economics spectrum (PES): under moderate drought, seedlings adopted an acquisitive strategy, where high SLA maximized light capture [28], high R/S prioritized water uptake [6,31], and WUE optimized water use [36]. Under severe drought, this acquisitive strategy broke down. Seedlings instead switched to a slow, conservative resource strategy that could not maintain continuous biomass accumulation [24].

In this model, antioxidant enzymes and osmotic regulatory factors also exhibited high explanatory power. It is evident that antioxidant and osmotic regulation are critically involved in the alleviation of plant physiological damage. N addition level had low explanatory power, indicating that it acts indirectly via modifying allocation, water use, and antioxidant capacity, modulated by drought intensity. The low explanatory power of Pn, Chl, and iWUE shows that photosynthetic traits alone cannot fully represent drought-adaptive strategies.

In conclusion, the hierarchical structure of key variables governing the responses of *Q. wutaishanica* seedlings to drought and nitrogen addition was clearly identified via random forest analysis. Quantitative references are therefore provided for the selection of evaluation indicators in future seedling drought-resistance research.

## 5. Conclusions

The responses of growth, photosynthesis and stress tolerance traits in *Q. wutaishanica* seedlings to varying drought intensities and nitrogen addition treatments were analyzed in this study. Intense drought was the dominant limiting factor, with significantly stronger inhibition than chronic drought. Nitrogen mitigation showed a clear threshold: effective under moderate drought but completely ineffective under intense drought. Random forest analysis identified biomass allocation and water-related physiological traits as core drivers of seedling responses. These findings provide a scientific basis for managing natural *Q. wutaishanica* forests, vegetation restoration, and rational N application. Moderate N input is recommended to improve seedling performance under chronic drought, whereas water supply should be prioritized over extra N under intense drought. This study provides empirical support for evaluating seedling regeneration and survival prospects amid progressive climate change. Admittedly, it is acknowledged that pot experiments cannot fully represent natural montane forest environments due to homogenized soil, confined root space and artificially controlled water and nitrogen regimes. Meanwhile, this study only included two drought intensities and one N level, and the effects of N-altered soil properties on seedlings were not fully clarified. Future work should incorporate continuous gradients of drought stress and nitrogen supply, incorporate warming treatments, and track temporal dynamics in soil physicochemical properties. Complementary field investigations are also required to verify our findings, enhance their extrapolation to natural forest ecosystems, and quantify long-term drought impacts on seedling development.

## Data Availability

All data generated or analyzed during this study are included in this published article [and its Supplementary Information files].

## Supplementary Materials

The following supporting information can be downloaded at https://www.mdpi.com/article/10.3390/biology15130991/s1, Table S1: Summary of mMeteorological Ddata for Jingyuan County.xls; Table S2: data(Drought).xlsx.
